# Cardiovascular risk and the COVID-19 pandemic: a population-based and case‒control studies

**DOI:** 10.1186/s12963-024-00338-w

**Published:** 2024-07-19

**Authors:** Małgorzata Chlabicz, Jacek Jamiołkowski, Marlena Dubatówka, Sebastian Sołomacha, Magdalena Chlabicz, Natalia Zieleniewska, Paweł Sowa, Anna Szpakowicz, Anna M. Moniuszko-Malinowska, Robert Flisiak, Marcin Moniuszko, Karol A. Kamiński

**Affiliations:** 1 Department of Population Medicine and Lifestyle Diseases Prevention, Medical University of Bialystok, ul. Waszyngtona 15B, Bialystok, 15-269 Poland; 2 Department of Invasive Cardiology, Medical University of Bialystok, Bialystok, Poland; 3 Department of Cardiology, Medical University of Bialystok, Bialystok, Poland; 4 Department of Infectious Diseases and Neuroinfections, Medical University of Bialystok, Bialystok, Poland; 5 Department of Infectious Diseases and Hepatology, Medical University of Bialystok, Bialystok, Poland; 6 Department of Allergology, Medical University of Bialystok, Bialystok, Poland

**Keywords:** COVID-19, Cardiovascular risk, Framingham Risk score, SCORE2-OP, LIFE-CVD

## Abstract

**Background:**

The coronavirus disease 2019 (COVID-19) pandemic is associated with increases in morbidity and mortality worldwide. The mechanisms of how SARS-CoV-2 may cause cardiovascular (CV) complications are under investigation. The aim of the study was to assess the impact of the COVID-19 pandemic on CV risk.

**Methods:**

These are single-centre Bialystok PLUS (Poland) population-based and case‒control studies. The survey was conducted between 2018 and 2022 on a sample of residents (*n* = 1507) of a large city in central Europe and patients 6–9 months post-COVID-19 infection (*n* = 126). The Systematic Coronary Risk Estimation 2 (SCORE2), the Systematic Coronary Risk Estimation 2-Older Persons (SCORE2-OP), the Cardiovascular Disease Framingham Heart Study and the LIFEtime-perspective model for individualizing CardioVascular Disease prevention strategies in apparently healthy people (LIFE-CVD) were used. Subsequently, the study populations were divided into CV risk classes according to the 2021 ESC Guidelines on cardiovascular disease prevention in clinical practice.

**Results:**

The study population consisted of 4 groups: a general population examined before (I, *n* = 691) and during the COVID-19 pandemic (II, *n* = 816); a group of 126 patients post-COVID-19 infection (III); and a control group matched subjects chosen from the pre-COVID-19 pandemic (IV). Group II was characterized by lower blood pressure, low-density lipoprotein cholesterol (LDL-c) and high-density lipoprotein cholesterol (HDL-c) values than group I. Group III differed from the control group in terms of lower LDL-c level. There was no effect on CV risk in the general population, but in the population post-COVID-19 infection, CV risk was lower using FS-lipids, FS-BMI and LIFE-CVD 10-year risk scores compared to the prepandemic population. In all subgroups analysed, no statistically significant difference was found in the frequency of CV risk classes.

**Conclusions:**

The COVID-19 pandemic did not increase the CV risk calculated for primary prevention. Instead, it prompted people to pay attention to their health status, as evidenced by better control of some CV risk factors. As the COVID-19 pandemic has drawn people’s attention to health, it is worth exploiting this opportunity to improve public health knowledge through the design of wide-ranging information campaigns.

## Introduction

The coronavirus disease 2019 (COVID-19) pandemic is associated with increases in morbidity and mortality worldwide. The precise mechanisms of how acute respiratory syndrome coronavirus 2 (SARS-CoV-2) may cause cardiovascular (CV) complications are under intensive investigation [1]. It has been shown to occur in large quantities in the heart during infection, which can lead to CV complications such as myocarditis, arrhythmias, cardiac arrest, acute myocardial injury, stress-induced cardiomyopathy, cardiogenic shock, and heart failure (HF) [2, 3]. Cardiac injury may be present in approximately 12–15% of hospitalized patients with COVID-19 [1, 4]. A few studies have examined CV outcomes in the postacute phase of COVID-19, but most have been limited to hospitalized individuals and to narrow the selection of CV outcomes [5, 6]. There are limited data on the impact of the COVID-19 pandemic on comprehensive CV risk assessment. Therefore, the aim of this study was to evaluate the impact of the COVID-19 pandemic on CV risk in the general population and in patients who recovered from COVID-19. Understanding the effects of SARS-CoV-2 infection on CV risk may help clinicians and health care professionals design research in a new epidemiological situation.

## Methods

### Study population

These are single-centre Bialystok PLUS (Poland) population-based and case‒control studies. The survey was conducted between 2018 and 2022 on a sample of residents of a large city in central Europe. Participants were randomly selected from among the city’s residents in such proportions to obtain a distribution of proportions similar to that of the city’s population [7]. In brief, each year (in the middle of the year, after June 30th), a pseudonymized list of Bialystok residents was obtained from the Bialystok City Hall. Then, the dataset was restricted to people aged 20–79, and categories based on gender and 5-year ranges (20–24, 25–29, etc.) were assigned, providing a total of 24 subcategories. From each subcategory separately, citizens were randomly selected in such numbers as to achieve a distribution of proportions similar to that of the city’s population. After sampling, the identifiers of the selected citizens were sent back to the City Hall to obtain their names and addresses in order to contact them. The selected citizens were invited to participate in the survey via a letter and encouraged to contact us by phone or email to schedule a visit. After some time, a second and even a third letter of invitation was sent to those who did not respond. Such a randomly selected number of citizens were examined over the course of a year. So the two study groups before and during COVID-19 were identically selected from Bialystok residents [7].

There were any exclusions, just some restrictions. Participants with an acute infectious disease or after surgery within the last six weeks were not examined, and they were encouraged to return to the study after this period. At the time of the examination, there were exclusions for individual procedures, e.g., pregnancy for dual energy X-ray absorptiometry (DEXA) and diabetes for oral glucose tolerance test (OGTT). During the COVID-19 pandemic, reverse transcription-polymerase chain reaction (RT‒PCR) was performed from nasopharyngeal swabs using the CFX96 Real-Time System (Bio-Rad) to exclude active COVID-19 infection. 20th March 2020, was considered the beginning of the COVID-19 pandemic in Poland. According to this date, the population was divided into two subgroups: before the COVID-19 pandemic (group I, *n* = 691) and during the COVID-19 pandemic (group II, *n* = 816). In the population during the COVID-19 pandemic, the anti-nucleocapsid-IgG antibody (anti-N IgG) were determined using electrochemiluminescence method (Cobas e411, ROCHE Diagnostic Ltd.,Rotkreuz, Switzerland). The analysis covered 727 individuals, among whom 445 were positive (61.2%).

**Fig. 1:**
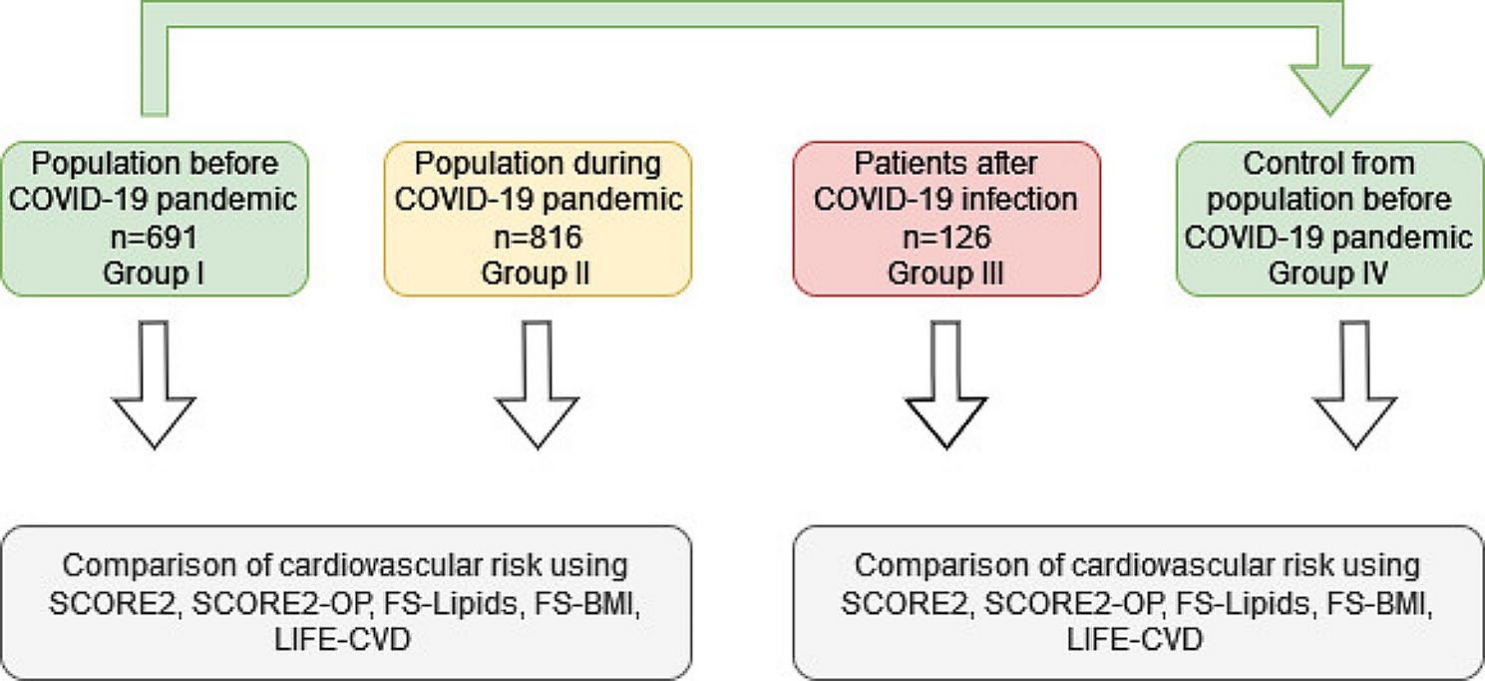
Flow chart of study construction

The subsequent analysed group was the independent group of patients who were hospitalized or under outpatient care due to symptomatic COVID-19 (group III, *n* = 126 patients). The diagnosis of SARS-CoV-2 infection was confirmed by RT‒PCR testing using the CFX96 Real-Time System (Bio-Rad) from nasopharyngeal swabs. This group was examined in detail in the 6–9 month period after the infection in accordance with the Bialystok PLUS Study protocol. These post-COVID-19 patients were compared (1:1) to 126 participants from the Bialystok PLUS population evaluated before the COVID-19 pandemic (group I). Matching was based on sex, age and body mass index (BMI) (group IV). The examinations of all populations were carried out according to the same procedures, in the same research centre and by the same trained staff. The participants in whom CV risk could not be calculated according to the European Society of Cardiology (ESC) Guidelines on cardiovascular disease prevention in clinical practice due to lack of data were excluded from the study [8]. A diagram of the survey design is shown in Fig. 1.

### Data collection

Data collection was conducted by trained staff. At the time of study entry, demographic characteristics and information on the participants’ medical history were collected from questionnaires. Peripheral intravenous fasting blood samples were collected at the time of visit in the morning after at least eight hours of fasting. The comprehensive assessment was performed as described in our previous articles [9–11]. High reproducibility of the tests was achieved by performing them according to validated standard operating procedures (SOPs). A list of parameters with the method and equipment used is presented in Table 1.

**Table 1:** Parameters, methods and equipment used in the study

Parameter	Method	Device
Fasting glucose and the 120 min glucose in oral glucose tolerance test (OGTT)	hexokinase method	Cobas s111, ROCHE Diagnostic Ltd., Rotkreuz, Switzerland
Haemoglobin A1c (HbA1c)	ion-exchange high-performance liquid chromatography (HPLC)	Bio-Rad, Hercules, CA, USA
Total cholesterol (TC)	enzymatic colorimetric method	Cobas s111, ROCHE Diagnostic Ltd., Rotkreuz, Switzerland
High-density lipoprotein cholesterol (HDL-c)	enzymatic colorimetric method	Cobas s111, ROCHE Diagnostic Ltd., Rotkreuz, Switzerland
Low-density lipoprotein cholesterol (LDL-c)	enzymatic colorimetric method	Cobas s111, ROCHE Diagnostic Ltd., Rotkreuz, Switzerland
Triglycerides (TG)	enzymatic colorimetric method	Cobas s111, ROCHE Diagnostic Ltd., Rotkreuz, Switzerland
Anti-nucleocapsid-IgG antibody (anti-N IgG)	electrochemiluminescence method (ECLIA)	Cobas e411, ROCHE Diagnostic Ltd., Rotkreuz, Switzerland
Creatinine	enzymatic colorimetric method	Cobas s111, ROCHE Diagnostic Ltd., Rotkreuz, Switzerland
Blood pressure (BP)	oscillometric method	Omron Healthcare Co. Ltd. MG Comfort device
Body composition	dual-energy X-ray absorptiometry (DEXA)	GE Healthcare, Chicago, IL, USA

The estimated glomerular filtration rate (eGFR) was calculated in line with the Modification of Diet in Renal Disease (MDRD) study equation. The albumin-to-creatinine ratio (ACR) is the method used to evaluate albuminuria in a urine sample. The ACR was calculated by dividing the albumin concentration in milligrams by the creatinine concentration in grams.

### Cardiovascular risk estimation

The Systematic Coronary Risk Estimation 2 (SCORE2) and the Systematic Coronary Risk Estimation 2-Older Persons (SCORE2-OP) [8], the Cardiovascular Disease Framingham Heart Study (FRS) [12] and the LIFEtime-perspective model for individualizing CardioVascular Disease prevention strategies in apparently healthy people (LIFE-CVD) [13] were used to calculate CV risk in primary prevention. The SCORE2 and SCORE2-OP systems assess the 10-year risk of fatal and nonfatal CV events (myocardial infarction, stroke) in apparently healthy people based on the following risk factors: age, sex, smoking, systolic blood pressure (BPs), and non-high-density lipoprotein cholesterol (non-HDL-c). The SCORE2 and SCORE2-OP were calculated, excluding participants who were prequalified in the high and very high CV risk classes, i.e., participants with previously diagnosed cardiovascular disease (CVD) (myocardial infarction – MI, ischemic heart disease – IHD, stroke, transient ischemic attack – TIA, peripheral arterial disease – PAD, significant plaque on carotid ultrasound > 50%), diabetes mellitus (DM) previously diagnosed or at the time of the study entry, chronic kidney disease (CKD) at the time of the study entry evaluated according to the mentioned guidelines based on the albumin/creatinine ratio (ACR) and estimated glomerular filtration rate (eGFR), familial hypercholesterolemia, and age younger than 40 years old. SCORE2 was calculated for those aged 40–69 years, and SCORE2-OP was calculated for those aged 70–89 years. The calculator for high CVD risk countries was used, as Poland belongs to this category. Subsequently, the study population was divided into CV risk classes according to the latest recommendation 2021 ESC Guidelines on cardiovascular disease prevention in clinical practice [8]. Initially, high- and very high-risk individuals were identified. Then, the previously calculated SCORE2 and SCORE2-OP values were used to categorize apparently healthy individuals. In this way, the entire population was categorized into low-to-moderate, high and very high risk classes [8].

The FRS predicted a 10-year risk of developing the first CVD event (IHD, stroke, PAD, or HF) using scores for body mass index (BMI) or lipids based on the following factors: age, smoking, DM, treated and untreated BPs, TC, HDL-c, or lipids replacing BMI [12]. Participants with previously diagnosed CVD (MI, IHD, stroke, TIA, PAD, significant plaque on carotid ultrasound > 50%) and younger than 30 years old or older than 74 years old were excluded from further analysis.

The LIFE-CVD calculates a 10-year risk of MI, stroke, or CV death; lifetime risk of MI, stroke, or CV death using the following factors: age, sex, smoking, geographic region, DM, parental history of MI prior to age 60, BPs, BMI, TC, HDL-c, and low-density lipoprotein cholesterol (LDL-c). Participants with previously diagnosed CVD (MI, IHD, stroke, TIA, PAD, significant plaque on carotid ultrasound > 50%) and younger than 35 years old were excluded from this analysis [13].

### Prevalence of CV risk-related diseases

The study also assessed known diseases associated with CV risk, such as hypertension, hypercholesterolemia, hypertriglyceridaemia and diabetes. The rates of people with known disease, appropriately treated disease, and newly diagnosed disease were assessed. Undiagnosed hypertension was considered when the participant without a history of hypertension had BPs ≥ 140 and/or BPd ≥ 90 mmHg. Well-controlled BP in patients diagnosed with hypertension was established when BPs < 130 and BPd < 80 mmHg below 65 years old, BPs < 140 and BPd < 80 mmHg 65–80 years old, and BPs < 150 and BPd < 80 mmHg over 80 years old were found. Undiagnosed hypercholesterolemia was considered when the participant had no history of hypercholesterolemia but had TC > 190 mg% or LDL-c > 100 mg% in the low-to-moderate CV class, > 70 mg% in the high CV class, and > 55 mg% in the very high CV class. A well-controlled lipid profile in patients with diagnosed hypercholesterolemia was established when LDL-c < 100 mg% in the low-to-moderate CV class, < 70 mg% in the high CV class, and < 55 mg% in the very high CV class. Undiagnosed triglyceridemia was established when TG > 150 mg% was found in the participant with a history of triglyceridemia. Well-controlled triglyceridemia in patients with diagnosed triglyceridemia was defined when TG < 150 mg%. Undiagnosed diabetes was stated when fasting glucose ≥ 126 mg/dl or OGGT 120 min. glucose ≥ 200 mg/dl or HbA1c ≥ 6,5% in participants with no history of diabetes. Well-controlled glucose in patients with diabetes was confirmed when HbA1c < 7.0% was found.

### Statistical analysis

The statistical analysis was performed using IBM SPSS Statistics 27.0 software (SPSS, Armonk, NY, USA) and Jupiter Notebook Python 3.9 statistical software (Anaconda distributor). Descriptive statistics for quantitative variables are presented as the mean and standard deviation (SD). The normality of distributions was assessed using the Shapiro‒Wilk test. Values of normally distributed compared by unpaired t test, whereas nonnormally distributed continuous data were compared by the Mann‒Whitney U test. Categorical variables are displayed as frequency distributions (n) and simple percentages (%). The chi2 test or Fisher’s exact test was used for the univariate comparison between the groups for categorical variables. Statistical significance was considered when *P* ≤ 0.05.

## Results

The study population consisted of 4 groups: a general population examined before (I, *n* = 691) and during the COVID-19 pandemic (II, *n* = 816); a group of 126 patients post-COVID-19 infection (III); and a control group matched subjects chosen from the pre-COVID-19 pandemic (IV). The studied populations did not differ by sex or age. The population during the COVID-19 pandemic (group II) was characterized by significantly lower systolic (BPs) and diastolic blood pressure (BPd), as well as LDL-c and HDL-c values, compared to the prepandemic population (group I). In contrast, this population had poorer renal function parameters (higher creatinine levels and lower eGFR) than the prepandemic COVID-19 population. The population after COVID-19 infection (group III) differed from the control group (group IV) in terms of lower LDL cholesterol levels. The characteristics of the studied populations are summarized in Table 2.

**Table 2:** Characteristics of the studied populations and comparison variables between them: general data, laboratory tests

Variable	Group I *N* = 691	Group II *N* = 816	*P*	Group III *N* = 126	Control group IV *N* = 126	*P*
Age, years	48.7 ± 15.4	49.3 ± 15.3	0.460	56.6 ± 12.5	58.5 ± 11.7	0.211
Male sex, n (%)	310 (44.9)	377 (44.9)	0.603	61 (48.4)	61 (48.4)	1.0
BPs, mmHg	124.9 ± 17.7	122.4 ± 17.3	0.006	127.0 ± 17.3	130.9 ± 19.2	0.096
BPd, mmHg	81.7 ± 10.1	79.8 ± 10.0	< 0.001	82.3 ± 10.6	84.7 ± 10.5	0.071
HR, bpm	71.8 ± 10.7	71.5 ± 10.7	0.594	68.8 ± 9.6	71.1 ± 10.3	0.065
Fasting glucose, mg/dL	103.5 ± 20.6	103.0 ± 18.4	0.178	106.5 ± 26.4	111.4 ± 20.6	0.102
OGTT 120 min glucose, mg/dL	125.2 ± 38.5	124.2 ± 36.8	0.145	-	-	-
HbA1c, %	5.5 ± 0.6	5.5 ± 0.6	0.520	5.6 ± 0.8	5.8 ± 0.6	0.073
TC, mg/dL	191.3 ± 41.4	192.6 ± 41.5	0.562	192.8 ± 45.6	195.3 ± 44.0	0.657
LDL-c, mg/dL	125.3 ± 37.5	120.3 ± 37.1	0.010	119.3 ± 40.0	130.5 ± 40.2	0.028
HDL-c, mg/dL	63.0 ± 17.2	60.0 ± 16.3	0.001	57.4 ± 15.2	59.0 ± 16.8	0.419
TG, mg/dL	112.7 ± 70.9	115.5 ± 82.4	0.471	128.2 ± 70.6	132.5 ± 66.4	0.615
Creatinine, µmol/L	69.1 ± 13.5	72.7 ± 25.8	0.001	81.4 ± 92.0	71.1 ± 13.1	0.215
eGFR, mL/min/1.73m^2^	101.3 ± 29.1	96.6 ± 21.4	0.001	93.2 ± 24.9	93.8 ± 18.4	0.627
ACR mg/g	7.3 ± 19.5	8.0 ± 13.0	0.427	11.3 ± 22.1	7.6 ± 17.4	0.136

The data is shown as n (%) or mean (SD). ACR, albumin*/*creatinine ratio; BP, blood pressure; BPd, diastolic blood pressure; BPs, systolic blood pressure; bpm, beats per min; eGFR, estimated glomerular filtration rate; HbA1 c, hemoglobin A1 c; HDL-c, high-density lipoprotein; HOMA-IR, homeostasis model assessment of insulin resistance; HR, heart rate; LDL-c, low-density lipoprotein; mmHg, millimetres of mercury; OGTT, oral glucose tolerance test; SD, standard deviation; TC, total cholesterol; TG, triglycerides

In terms of anthropometric parameters, the population during the pandemic (group II) was characterized by greater hip and thigh circumference, total fat mass and lower WHR compared to group I. The population after COVID-19 infection (group III) differed from the control group (group IV) only in having a lower waist-hip ratio (WHR). The exact data are shown in Table 3.

**Table 3:** Characteristics of the studied populations and comparison variables between them: anthropometric measurements, body composition analysis

Variable	Group I *N* = 691	Group II *N* = 816	*P*	Group III *N* = 126	Control group IV *N* = 126	*P*
Height, cm	170.1 ± 10.1	170.5 ± 9.7	0.487	168.8 ± 9.8	167.8 ± 10.0	0.429
Body mass, kg	77.6 ± 16.4	79.1 ± 16.6	0.090	84.2 ± 16.8	85.5 ± 16.2	0.556
BMI, kg/m^2^	26.8 ± 4.8	27.2 ± 5.0	0.121	29.5 ± 5.2	30.3 ± 4.9	0.224
Waist, cm	87.4 ± 13.5	88.7 ± 13.5	0.055	94.7 ± 12.6	96.3 ± 12.6	0.319
Hips, cm	99.1 ± 9.6	102.7 ± 9.0	< 0.001	105.7 ± 10.1	104.5 ± 10.3	0.367
Thigh, cm	58.5 ± 5.9	59.6 ± 5.9	< 0.001	59.9 ± 8.3	60.1 ± 6.4	0.772
WHR	0.88 ± 0.10	0.86 ± 0.10	< 0.001	0.90 ± 0.09	0.92 ± 0.09	0.022
Total fat mass, kg	26.0 ± 9.0	27.0 ± 9.6	0.044	30.3 ± 10.1	31.9 ± 9.5	0.196
FMI, kg/m^2^	9.1 ± 3.5	9.4 ± 3.6	0.129	10.8 ± 3.8	11.5 ± 3.7	0.137
Total lean mass, kg	49.2 ± 10.8	50.0 ± 10.5	0.173	51.5 ± 10.6	51.0 ± 10.3	0.678
LMI, kg/m^2^	16.8 ± 2.4	17.0 ± 2.4	0.099	17.9 ± 2.4	17.9 ± 2.2	0.921
Android fat mass, kg	2.4 ± 1.2	2.5 ± 1.3	0.094	3.1 ± 1.3	3.3 ± 1.2	0.152
Gynoid fat mass, kg	4.0 ± 1.4	4.1 ± 1.5	0.157	4.5 ± 1.6	4.6 ± 1.6	0.387
Visceral mass, kg	1.2 ± 1.0	1.3 ± 1.0	0.089	1.8 ± 1.1	1.9 ± 1.0	0.496
A/G fat ratio	0.59 ± 0.24	0.61 ± 0.24	0.266	0.69 ± 0.23	0.73 ± 0.24	0.187

The data is shown as n (%) or mean (SD). A, android; BMI, body mass index; FMI, fat mass index; G, gynoid; LMI, lean mass index; WHR, waist-hip ratio

**Fig. 2:**
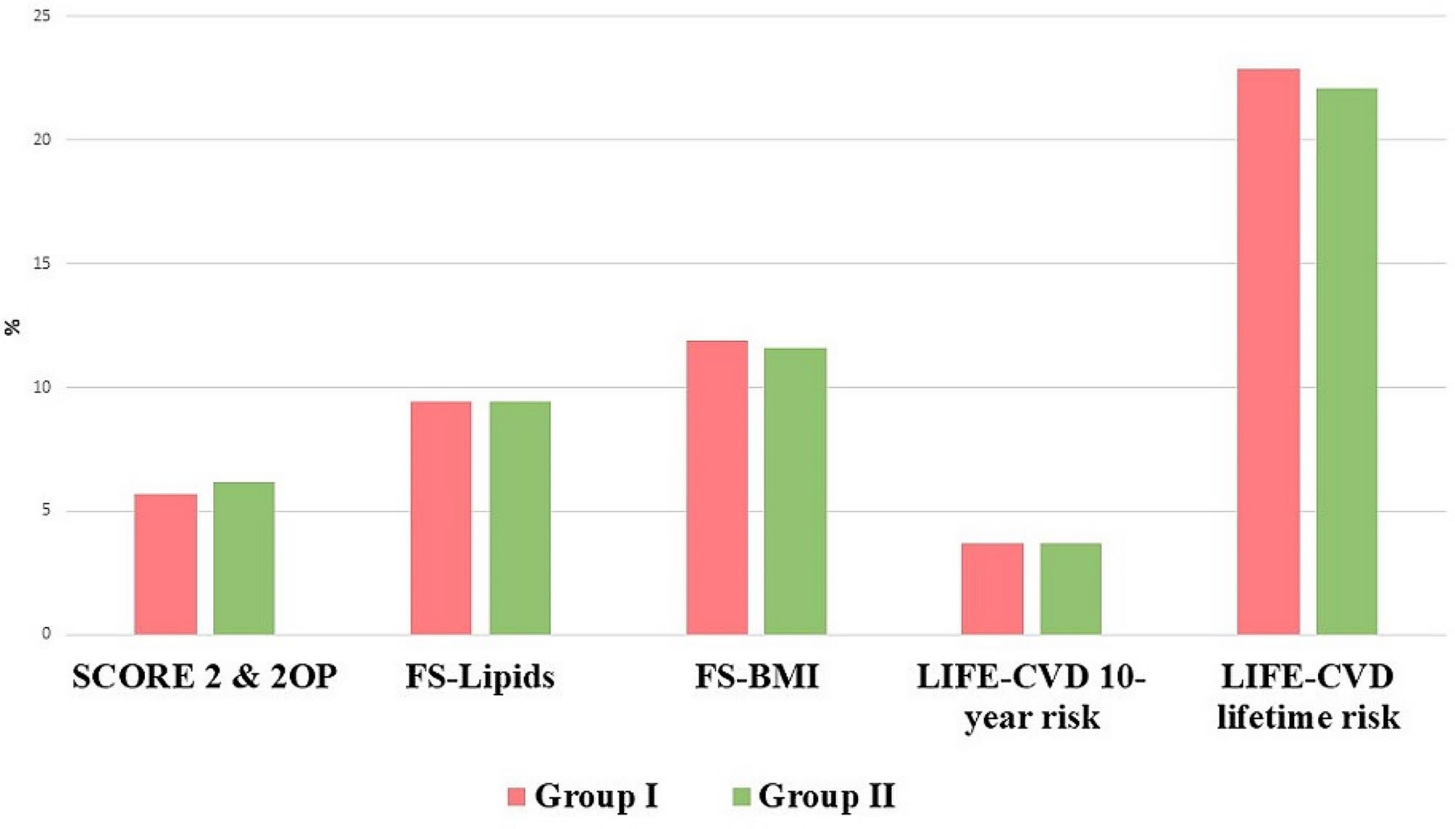
The value of cardiovascular risk of the population before the COVID-19 pandemic (group I) and during the COVID-19 pandemic (group II)

**Fig. 3:**
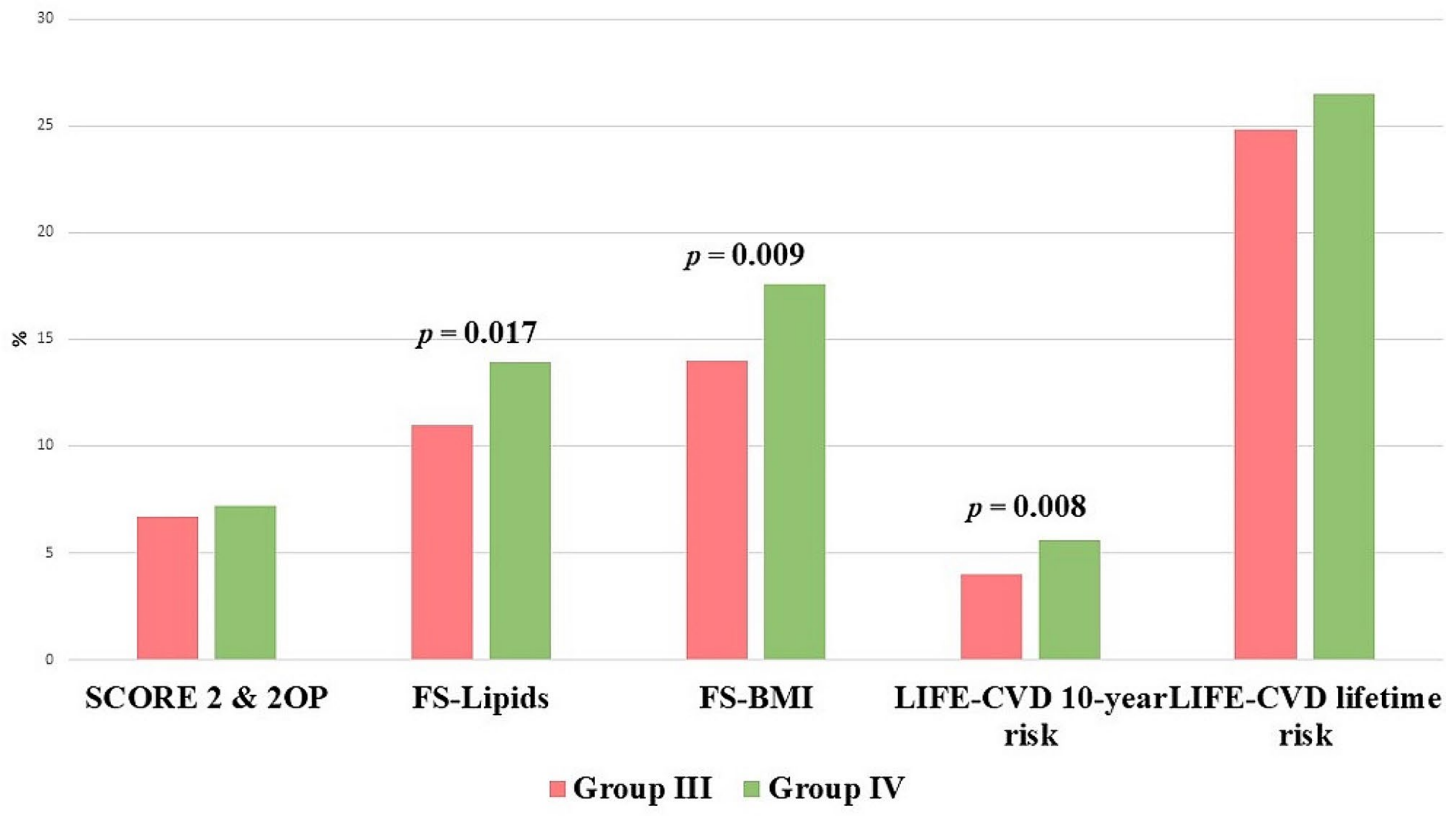
The cardiovascular risk of the population after COVID-19 infection (group III) and the control group before the COVID-19 pandemic (group IV)

In the analysis of CV risk using different primary prevention scales, no statistically significant differences were found between the population before (group I) and during the COVID-19 pandemic (group II) (Table 4; Fig. 2). In contrast, the population after COVID-19 infection (group III) had a significantly lower CV risk using FS-Lipids, FS-BMI and LIFE-CVD 10-year risk than the control group (group IV) (Table 4; Fig. 3). In all subgroups analysed, no difference was found in the frequency of CV risk classes (Table 4).

**Table 4:** The value of cardiovascular risk and the frequency of CV risk classes of the studied populations

Variable	Group I *N* = 691	Group II *N* = 816	*P*	Group III *N* = 126	Control group IV *N* = 126	*P*
SCORE 2 & 2OP, %	5.7 ± 5.2	6.2 ± 5.6	0.203	6.7 ± 4.9	7.2 ± 5.3	0.553
FS-Lipids, %	9.4 ± 9.0	9.4 ± 9.0	0.918	11.0 ± 8.6	13.9 ± 9.8	0.017
FS-BMI, %	11.9 ± 10.3	11.6 ± 10.3	0.678	14.0 ± 9.4	17.6 ± 10.6	0.009
LIFE-CVD 10-year risk, %	3.7 ± 4.1	3.7 ± 3.6	0.880	4.0 ± 3.1	5.6 ± 5.7	0.008
LIFE-CVD lifetime risk, %	22.9 ± 10.2	22.1 ± 9.7	0.199	24.8 ± 9.8	26.5 ± 11.3	0.228
CVD-free life-expectancy	87.8 ± 7.5	87.7 ± 7.2	0.879	88.7 ± 5.3	87.7 ± 5.7	0.180
Low to moderate CV risk class^*^	439 (63.5)	482 (59.1)	0.189	44 (34.9)	47 (37.3)	0.916
High CV risk class^*^	166 (24.0)	214 (26.2)		51 (40.5)	50 (39.7)	
Very-high CV risk class^*^	86 (12.4)	120 (14.7)		31 (24.6)	29 (23.0)	

The data is shown as n (%), mean (SD). BMI, body mass index; CV, cardiovascular; CVD, cardiovascular disease; FRS, Framingham Risk Score; LIFE-CVD, LIFEtime-perspective model for individualizing CardioVascular Disease prevention strategies in apparently healthy people; SCORE 2OP, Systematic Coronary Risk Estimation 2 and Systematic Coronary Risk Estimation 2-Older Persons risk; SD, standard deviation.

^*^The study population was divided into CV risk classes in line with 2021 ESC Guidelines on cardiovascular disease prevention in clinical practice. Eur J Prev Cardiol, 2021

A comparison of medical history in terms of CVD between the analysed populations showed a significantly higher rate of history of atrial fibrillation (AF) and history of hypercholesterolaemia in the population during the COVID-19 pandemic (group II) compared to group I, while the rates of unrecognized hypercholesterolemia and hypertriglyceridaemia were lower, and lipid disorders were better controlled in group II. In the population after COVID-19 infection (group III), there was also better control of lipid disorders but worse control of glucose disorders. Detailed information can be found in Table 5.

**Table 5:** Medical history of the studied populations and comparison variables between subgroups

Medical history	Group I *N* = 691	Group II *N* = 816	*P*	Group III *N* = 126	Control group IV *N* = 126	*P*
Ever smoked cigarettes	395 (57.6)	468 (57.6)	0.995	66 (52.4)	82 (65.1)	0.041
Currently smoking	132 (19.3)	154 (19.1)	0.905	8 (6.3)	17 (13.5)	0.058
History of ischemic heart disease	21 (3.0)	35 (4.3)	0.199	7 (5.6)	7 (5.6)	1.000
History of heart failure	9 (1.3)	14 (1.7)	0.513	2 (1.6)	2 (1.6)	1.000
History of stroke	7 (1.0)	9 (1.1)	0.863	3 (2.4)	3 (2.4)	1.000
History of peripheral artery disease	6 (0.9)	11 (1.3)	0.378	3 (2.4)	3 (2.4)	1.000
History of atrial fibrillation	18 (2.6)	40 (4.9)	0.021	7 (5.6)	5 (4.0)	0.554
History of hypertension	196 (28.4)	237 (29.1)	0.760	62 (49.2)	62 (49.2)	1.000
Well-controlled BP in patients diagnosed with hypertension^*^	50 (25.5)	80 (33.8)	0.077	20 (32.3)	20 (32.3)	1.000
Undiagnosed hypertension	96 (13.9)	94 (11.5)	0.172	12 (9.5)	19 (15.1)	0.179
History of hypercholesterolemia	194 (28.1)	308 (37.8)	< 0.001	64 (50.8)	53 (42.1)	0.165
Well-controlled lipid profile in patients with diagnosed hypercholesterolemia^†^	16 (8.2)	45 (14.6)	0.002	11 (17.1)	3 (5.7)	0.027
Undiagnosed hypercholesterolemia^‡^	382 (55.3)	362 (44.4)	< 0.001	54 (42.9)	64 (50.8)	0.207
History of triglyceridemia	104 (15.1)	156 (19.1)	0.036	38 (30.2)	32 (25.4)	0.399
Well-controlled triglyceridemia in patients with diagnosed triglyceridemia^§^	62 (59.6)	97 (62.2)	0.065	23 (18.4)	18 (14.3)	0.378
Undiagnosed triglyceridemia^e^	86 (12.4)	108 (13.3)	0.642	19 (15.1)	21 (16.7)	0.730
History of diabetes	43 (6.2)	54 (6.6)	0.756	8 (6.5)	18 (14.3)	0.617
Well controlled glucose in patients diagnosed with diabetes ^||^	29 (67.4)	34 (63.0)	0.977	3 (57.5)	14 (77.8)	0.006
Undiagnosed diabetes ^**^	45 (6.5)	48 (5.9)	0.617	12 (9.6)	25 (19.8)	0.022
Antihypertensive drugs	206 (29.8)	239 (29.3)	0.825	56 (44.4)	66 (52.4)	0.207
Anticholesterol drugs	95 (13.7)	122 (15.0)	0.508	30 (23.8)	34 (27.0)	0.563
Antidiabetic drugs	44 (6.4)	62 (7.6)	0.352	9 (7.1)	17 (13.5)	0.098

The data are shown as n (%). BP, blood pressure; BPs, systolic blood pressure; BPd, diastolic blood pressure; CV, cardiovascular; HbA1c, hemoglobin A1c; HDL, high-density lipoprotein; LDL, low-density lipoprotein; OGTT, oral glucose tolerance test; TC, total cholesterol; TG, triglycerides; ^*^BPs < 130 and BPd < 80 mmHg below 65 years old, BPs < 140 and BPd < 80 mmHg 65–80 years old, BPs < 150 and BPd < 80 mmHg over 80 years old; ^†^LDL-c < 116 mg% in low CV class, < 100 mg% in moderate CV class, < 70 mg% in high CV class, < 55 mg% in very-high CV class; ^‡^TC > 190 mg% or LDL-c > 116 mg% in low CV class, > 100 mg% in moderate CV class, > 70 mg% in high CV class, > 55 mg% in very-high CV class; ^§^TG < 150 mg%; ^e^TG>150 mg%; ^||^HbA1c < 7.0% ^**^ Fasting glucose ≥ 126 mg/dl or OGGT 120 min. glucose ≥ 200 mg/dl or HbA1c ≥ 6,5%

## Discussion

This combined population-based and case‒control survey calculates CV risk using different methods in the population during the COVID-19 pandemic and recovery from COVID-19. The conducted analysis did not reveal significant differences in the CV risk calculated for primary prevention in the populations during the COVID-19 pandemic compared to the prepandemic population. In contrast, in the population that had symptomatic COVID-19 infection, the CV risk was lower. Eventually, no differences in the prevalence of CV risk classes were found in any of the subgroups analysed.

Many studies have shown that increased CV risk adversely affects the course of SARS-CoV-2 infection [14–17], i.e., AH with a primary pooled relative risk estimate of 3.08 (95% CI 2.33–4.07), DM with a primary pooled relative risk estimate of 3.55 (95% CI 2.56–4.93), and CVD with a primary pooled relative risk estimate of 5.05 (95% CI 4.36–5.85) [14]. Moreover, in COVID-19 patients, AF was associated with a 4-fold higher risk of death [18]. Postacute cardiovascular manifestations of COVID-19 have been described. Garcia-Zamora et al. [19] conducted a systematic review and meta-analysis to examine the prevalence of cardiac arrhythmias detected by electrocardiography (ECG) and their relationships with adverse outcomes in patients with COVID-19. They showed that QTc prolongation, ST-segment deviation, and various cardiac arrhythmias were observed in patients hospitalized with COVID-19 and were associated with a worse prognosis. Xiong et al. [20] described the prevalence, nature and risk factors for the main sequelae in COVID-19 survivors who had been discharged from the hospital for more than 3 months. There was no difference in the incidence of hypertension, DM, CVD, or CKD compared to the COVID-19-free control group. In contrast, Ayoubkhani et al. [5] showed that during a 140-day follow-up period, almost one-third of those who were discharged from the hospital after acute COVID-19 were readmitted, and more than 1 in 10 died after hospital discharge. The incidence of respiratory disease, diabetes and CVD was significantly elevated in patients with COVID-19 compared to the general population. Likewise, Xie et al. [6] showed that individuals with COVID-19 were at increased risk of incident CVD spanning several categories, including arrhythmias, cerebrovascular disorders, ischemic and nonischemic heart disease, HF, pericarditis, myocarditis, and thromboembolic disease. These results provide evidence that the risk and 1-year burden of CVD in acute COVID-19 survivors is substantial.

The CV risks after COVID-19 estimated in primary prevention have not yet been comprehensively described, and only the association of CV risk with the course of COVID-19 infection has been described previously. Mozzini et al. [21] evaluated the association between CV scoring systems (FRS, atherosclerotic cardiovascular disease score: ASCVD) and chest X ray (CXR) examination in 50 COVID-19 patients. Patients who died had a higher FRS than survivors. They found a strong correlation between CXR severity and FRS and ASCVD – high CV risk patients had consolidations more frequently. Similarly, Warren-Gash et al. [22] divided the historical population into categories: existing CVD, elevated risk cardiovascular risk (defined by QRISK3 score ≥ 10%) and low risk (QRISK3 score < 10%). The authors showed distinct differences in the incidence of all serious effects of COVID-19 by CV risk profile.

To the best of our knowledge, this is the first study to assess CV risk in the population during the COVID-19 pandemic and recovered from COVID-19 using validated and known tools for primary prevention risk assessment. The current study found that the COVID-19 pandemic did not increase the CV risk calculated for primary prevention. Better control of some CV risk factors, such as BP or hyperlipidaemia, which are part of CV risk scales, was reported. The contact with healthcare due to COVID-19 probably increased the number of blood labs, and this may also be an effect of a preventive “40+” program, which was introduced on 1 July 2021 in the Polish healthcare system. The “40+” program includes a packet of laboratory measurements, including cholesterol concentrations, available free of charge to all adults above the age of 40. These situations may have had an impact on improving health literacy (HL).

HL is a protective factor for certain chronic diseases, and its role in the COVID-19 pandemic has been investigated by Tao et al. [23]. They showed that people with higher HL were more likely to have adequate knowledge of COVID-19 than people with limited HL (OR = 3.473, 95% CI = 2.974–4.057, *P* < 0.001), more positive attitudes and more active behaviour. The literature has studied the relationship between HL and self-care for CVD, which includes adherence to treatment recommendations, monitoring of symptoms, and early response to symptoms when they appear [24]. Adequate self-care has been shown to improve CV outcomes, including improved quality of life, reductions in hospitalizations and mortality [24, 25]. Therefore, we conclude that the survival of COVID-19 prompted people to pay attention to their state of health, as evidenced by improved control of hyperlipidaemia.

However, it should be emphasized that the current study found a significantly higher rate of history of AF and poorer renal function in the population during the COVID-19 pandemic than in the prepandemic population. These parameters are not considered in the calculators for primary CV risk, but eGFR and ACR are parameters used in stratifying CV risk classes according to the latest ESC guidelines [8]. This resulted in an increase in the percentage of high and very high CV risk classes at the expense of low to moderate CV classes in both analysed groups. These changes were not statistically significant but deserve attention and further careful observation. Schiffl H et al. [26] showed that patients who have survived COVID-19 face an increased risk of worse kidney outcomes in the postacute phase of the disease and may predispose surviving patients to CKD, independent of clinically apparent acute kidney injury (AKI). Our study is consistent with previous observations.

We must emphasize that the CV risk was calculated during the pandemic, whereas the development of various risk factors as well as the progression of atherosclerosis is a lengthy process; therefore, the lack of differences in CV risk between the population just before and during the pandemic does not implicate the lack of such an effect after some time and should be analysed in future studies.

## Conclusions

The COVID-19 pandemic did not increase the CV risk calculated for primary prevention. Instead, it prompted people to pay attention to their health status, as evidenced by better control of some CV risk factors. As the COVID-19 pandemic has drawn people’s attention to health, it is worth exploiting this opportunity to improve public health knowledge through the design of wide-ranging information campaigns.

### Study limitations

This study has some limitations. This study is limited by a sample from one region, which is an urban environment. This is a single-centre study with a limited number of samples and a short observation period. Despite the limited number of cases, the findings from this study are novel and can be the basis of future studies. A enormous advantage of the study is that the examination of the general population before the pandemic, during the pandemic and of patients after COVID-19 was carried out according to the same procedures, in the same study centre and by the same trained staff.

## Data Availability

The datasets are not publicly available because the individual privacy of the participants should be protected. However, data are available from the corresponding author upon reasonable request.

## Abbreviations

ACR: Albumin-to-creatinine ratio
AF: Atrial fibrillation
ASCVD: Atherosclerotic cardiovascular disease score
BMI: Body mass index
BP: Blood pressure
CKD: Chronic kidney disease
COVID-19: Coronavirus disease 2019
CV: Cardiovascular
CXR: Chest X ray
CVD: Cardiovascular disease
DEXA: Dual energy X-ray absorptiometry
DM: Diabetes mellitus
eGFR: Estimated Glomerular Filtration Rate
ECG: Electrocardiography
ESC: European Society of Cardiology
FRS: Cardiovascular Disease Framingham Heart Study
HbA1c: Haemoglobin A1c
HDL-c: High-density lipoprotein cholesterol
HF: Heart failure
IHD: Ischemic heart disease
HL: Health literacy
LDL-c: Low-density lipoprotein cholesterol
LIFE-CVD: LIFE time-perspective model for individualizing cardiovascular disease prevention strategies in apparently healthy people
MDRD: Modification of Diet in Renal Disease study equation
MI: Myocardial infarction
OGTT: Oral glucose tolerance test
PAD: Peripheral arterial disease
RT‒PCR: Reverse transcription-polymerase chain reaction
SARS-CoV-2: Acute respiratory syndrome coronavirus 2
SCORE2: Systematic Coronary Risk Estimation 2
SCORE2-OP: Systematic Coronary Risk Estimation 2-Older Persons
SOP: Standard operating procedure
TC: Total cholesterol
TG: Triglycerides
TIA: Transient ischemic attack
